# Complete blood count and C-reactive protein to predict positive blood culture among neonates using machine learning algorithms

**DOI:** 10.1016/j.clinsp.2022.100148

**Published:** 2022-12-08

**Authors:** Felipe Yu Matsushita, Vera Lúcia Jornada Krebs, Werther Brunow de Carvalho

**Affiliations:** Department of Pediatrics, Neonatology Division, Faculdade de Medicina da Universidade de São Paulo, São Paulo, SP, Brazil

**Keywords:** Critical care, Neonatology, Artificial intelligence, Machine learning, Sepsis, ML, Machine Learning, AUROC, Area Under the Receiver Operating Characteristics, CRP, C-reactive Protein, CBC, Complete Blood Count, MCV, Mean Corpuscular Volume, MCH, Mean Corpuscular Hemoglobin, NLR, Neutrophil/Lymphocyte Ratio, MLR, Monocyte/Lymphocyte Ratio, PLR, Platelet/Lymphocyte Ratio, DNI, Delta Neutrophil Index

## Abstract

**Purpose:**

The authors aimed to develop a Machine-Learning (ML) algorithm that can predict positive blood culture in the neonatal intensive care unit, using complete blood count and C-reactive protein values.

**Methods:**

The study was based on patients’ electronic health records at a tertiary neonatal intensive care unit in São Paulo, Brazil. All blood cultures that had paired complete blood count and C-reactive protein measurements taken at the same time were included. To evaluate the machine learning model's performance, the authors used accuracy, Area Under the Receiver Operating Characteristics (AUROC), recall, precision, and F1-score.

**Results:**

The dataset included 1181 blood cultures with paired complete blood count plus c-reactive protein and 1911 blood cultures with paired complete blood count only. The f1-score ranged from 0.14 to 0.43, recall ranged from 0.08 to 0.59, precision ranged from 0.29 to 1.00, and accuracy ranged from 0.688 to 0.864.

**Conclusion:**

Complete blood count parameters and C-reactive protein levels cannot be used in ML models to predict bacteremia in newborns.

## Highlights

• It can take days to get the result of blood culture.
• CBC and CRP are readily available exams and could be used to predict blood culture.
• ML algorithms based on CBC and CRP couldn't predict neonatal blood culture positivity.

## Introduction

Bacteremia is a deadly condition in adults, with mortality rates ranging from 15.4 to 27.7%.^1^ The global mortality of neonatal sepsis is estimated at 17.6%.^2^ Early diagnosis and treatment are therefore essential for reducing morbimortality. The gold standard for diagnosing bacteremia is blood culture analysis, which might take hours or days to get a result.^3^ Additionally, neonatal sepsis signs and symptoms may be mild and challenging to distinguish from non-infectious conditions.^4^ Therefore, in order to anticipate bacteremia detection prior to blood culture end-result, laboratory biomarkers such as Complete Blood Count (CBC), procalcitonin, and C-Reactive Protein (CRP) have been adopted into clinical practice.

Both CBC parameters that relate to neonatal sepsis, such as the immature to total neutrophil ratio and CRP, have wide-ranging diagnostic accuracies.^4^^,^^5^ As a result, there aren't any diagnostic indicators available right now that are sensitive and specific enough to decide whether or not to withhold antibiotics in neonatal sepsis.

It is challenging to translate the findings of clinical research into clinical practice because of the complexity of medicine. Predictive models utilizing machine learning algorithms are becoming increasingly popular in this context. Machine learning models are being used to forecast a wide range of illnesses, including acute kidney injury and heart failure.^6^

The two most frequent laboratory tests performed on individuals with suspected sepsis are CBC and CRP.^7^ However, the analysis of CBC and CRP in machine-learning models in newborns has not been explored yet. Procalcitonin appears to be more reliable than CRP in predicting bacteremia, but it is more expensive.^8^

The authors created ML models to analyze the viability of using CBC and CRP to predict and identify early bacteremia in neonates. The authors also investigated the capacity of ML to predict declines when CRP is excluded.

## Material and methods

### Study population

This retrospective study was conducted at a single-center tertiary neonatal intensive care unit in São Paulo, Brazil. Data from all newborns admitted to the neonatal intensive care unit who were born between 2018 and 2021 were examined. All data were obtained from electronic medical records and uploaded to a data repository. The study protocol was approved by the institutional ethics committee *– Comite de Ética do Hospital das Clínicas da Faculdade de Medicina da Universidade de São Paulo* (CAAE 15762719.6.0000.0068) and waived informed consent. All blood cultures with paired CBC and CRP measurements that were taken simultaneously were included. The samples with CBC and CRP has taken on the same blood culture day but not at the same time were excluded from the study.

### Predictive parameters

A total of 25 feasible parameters were included in the machine-learning algorithms. These parameters included hemoglobin, hematocrit, MCV (Mean Corpuscular Volume), MCH (Mean Corpuscular Hemoglobin), MCHC (Mean corpuscular hemoglobin concentration), leukocytes, neutrophils (%), neutrophils (absolute count) neutrophil left shift (%), neutrophil left shift (absolute count), eosinophils (%), eosinophils (absolute count), basophils (%), basophils (absolute count), lymphocytes (%), lymphocytes (absolute count), monocytes (%), monocytes (absolute count), platelets, NLR (Neutrophil/Lymphocyte Ratio), MLR (Monocyte/Lymphocyte Ratio), PLR (Platelet/Lymphocyte Ratio), DNI (Delta Neutrophil Index), CRP (C-Reactive Protein) and Lymphocyte to CRP ratio. The authors did not include demographic data as predictive parameters due to the high rate of missing data on these parameters.

### Feature selection

The authors created a total of 9 different machine learning models, with the main distinction between them being the subset of variables that each model covered. In models 1 to 5, CBC parameters and CRP values were used. Only CBC variables were used in models 6 to 9.

### Machine learning model development

For each of the 9 models, the authors compared the performance of fourteen machine learning techniques to predict a positive blood culture: Random Forest Classifier, Extra Trees Classifier, Logistic Regression, Ridge Classifier, Linear Discriminant Analysis, Light Gradient Boosting Machine, Gradient Boosting Classifier, Extreme Gradient Boosting, K Neighbors Classifier, Ada Boost Classifier, Decision Tree Classifier, Naïve Bayes, SVM – linear kernel, Quadratic Discriminant Analysis. Patient datasets were randomly divided into two subsets: a training subset (70%) for hyperparameter tuning to create a plausible model, and a validation subset (30%) for testing the model's performance. In the training phase, the authors selected the model with the highest accuracy and performed the hyperparameter tuning only on this model.

### Machine learning models

#### Model 1

The authors included all 25 parameters in the machine learning models. Random Forest Classifier achieved the highest accuracy. After tuning, the following hyperparameters were used: bootstrap=True, ccp_alpha=0.0, class_weight={}, criterion='gini', max_depth=11, max_features='sqrt', max_leaf_nodes=None, max_samples=None, min_impurity_decrease=0.005, min_impurity_split=None, min_samples_leaf=4, min_samples_split=9, min_weight_fraction_leaf=0.0, n_estimators=150, n_jobs=-1, oob_score=False, random_state=142, verbose=0, warm_start=False).

#### Model 2

Only variables that were statistically significant in univariate analysis were included (Hemoglobin, Hematocrit, MCV, MCH, MCHC, Neutrophils (%), Neutrophils absolute count, Left shift (%), Left shift absolute count, NLR, PLR, MLR, DNI, Basophils absolute count, Lymphocytes (%), Lymphocytes absolute count, CRP, Platelet, and Lymphocyte/CRP ratio). The following hyperparameters were used: bootstrap=False, ccp_alpha=0.0, class_weight='balanced', criterion='entropy', max_depth=8, max_features='log2′, max_leaf_nodes=None, max_samples=None, min_impurity_decrease=0, min_impurity_split=None, min_samples_leaf=5, min_samples_split=7, min_weight_fraction_leaf=0.0, n_estimators=240, n_jobs=-1, oob_score=False, random_state=142, verbose=0, warm_start=False.

#### Model 3

In this model, the authors used the Boruta SHAP feature selection algorithm to select features into machine learning models (Hematocrit, Neutrophils %, Lymphocyte to CRP ratio, MCH, Shift, Platelet, PLR, MCV, Monocyte, C-reactive protein). This algorithm combines the Boruta algorithm (which identifies only features that have importance to the desired outcome) and SHAP (Shapley Additive exPlanations) technique(9). The following hyperparameters were used: bootstrap=False, ccp_alpha=0.0, class_weight='balanced', criterion='entropy', max_depth=8, max_features='log2′, max_leaf_nodes=None, max_samples=None, min_impurity_decrease=0, min_impurity_split=None, min_samples_leaf=5, min_samples_split=7, min_weight_fraction_leaf=0.0, n_estimators=240, n_jobs=-1, oob_score=False, random_state=142, verbose=0, warm_start=False.

#### Model 4

In this model, the authors included features according to experts’ opinions in machine learning models (Neutrophils left shift (%), DNI, Lymphocytes (%), CRP, and platelet). Linear Discriminant Analysis achieved the highest accuracy. After tuning, the following hyperparameters were used: LinearDiscriminantAnalysis (n_components=None, priors=None, shrinkage=0.3, solver='eigen', store_covariance=False, tol=0.0001).

#### Model 5

In this model, the authors activated PyCaret's feature_selection (it uses a combination of feature selection techniques to select the subset of features that are most important for modeling) and remove_multicollinearity (which drop features that are highly correlated with each other) parameters.^10^ The extra Trees Classifier model achieved the highest accuracy. The following hyperparameters were used: bootstrap=False, ccp_alpha=0.0, class_weight='balanced', criterion='entropy', max_depth=6, max_features='sqrt', max_leaf_nodes=None, max_samples=None, min_impurity_decrease=0.002, min_impurity_split=None, min_samples_leaf=4, min_samples_split=5, min_weight_fraction_leaf=0.0, n_estimators=70, n_jobs=-1, oob_score=False, random_state=142, verbose=0, warm_start=False.

#### Model 6

In this model, the authors included all 23 parameters in machine learning models. Gradient Boosting Classifier achieved the highest accuracy. The following hyperparameters were used: ccp_alpha=0.0, criterion='friedman_mse', init=None, learning_rate=0.05, loss='deviance', max_depth=1, max_features=1.0, max_leaf_nodes=None, min_impurity_decrease=0.2, min_impurity_split=None, min_samples_leaf=3, min_samples_split=4, min_weight_fraction_leaf=0.0, n_estimators=150, n_iter_no_change=None, presort='deprecated', random_state=142, subsample=0.85, tol=0.0001, validation_fraction=0.1, verbose=0, warm_start=False).

#### Model 7

Only variables that were statistically significant in univariate analysis were included. Extra Trees Classifier achieved the highest accuracy. The following hyperparameters were used: bootstrap=False, ccp_alpha=0.0, class_weight='balanced', criterion='entropy', max_depth=8, max_features='log2′, max_leaf_nodes=None, max_samples=None, min_impurity_decrease=0, min_impurity_split=None, min_samples_leaf=5, min_samples_split=7, min_weight_fraction_leaf=0.0, n_estimators=240, n_jobs=-1, oob_score=False, random_state=142, verbose=0, warm_start=False.

#### Model 8

In this model, the authors used Boruta SHAP feature selection algorithm^9^ to select features into machine learning models (Monocytes/Lymphocytes ratio, Hemoglobin, Neutrophils/Lymphocytes ratio, Monocytes (%), CHM, Platelets, Neutrophils (%), Platelet/Lymphocytes ratio, CVM, Hematocrit). Linear Discriminant Analysis achieved the highest accuracy. After tuning, the following hyperparameters were used: n_components=None, priors=None, shrinkage=0.4, solver='eigen', store_covariance=False, tol=0.0001.

#### Model 9

In this model, the authors activated PyCaret's feature_selection and remove_multicollinearity.^10^ The extra Trees Classifier model achieved the highest accuracy. The following hyperparameters were used: bootstrap=False, ccp_alpha=0.0, class_weight='balanced', criterion='entropy', max_depth=8, max_features='log2′, max_leaf_nodes=None, max_samples=None, min_impurity_decrease=0, min_impurity_split=None, min_samples_leaf=5, min_samples_split=7, min_weight_fraction_leaf=0.0, n_estimators=240, n_jobs=-1, oob_score=False, random_state=142, verbose=0, warm_start=False.

### Statistical analysis

Continuous variables were tested for normality using the Kolmogorov-Smirnov test. To compare laboratory parameters between positive and negative blood culture the authors used the Mann-Whitney test for continuous variables. All analyses were conducted using Python version 3.8.2 and the Pycaret python library.^10^ All patients with missing data were excluded from the study. The code is available at: https://github.com/fymatsushita/bloodculture.

### Performance measures

**Fig. 1:**
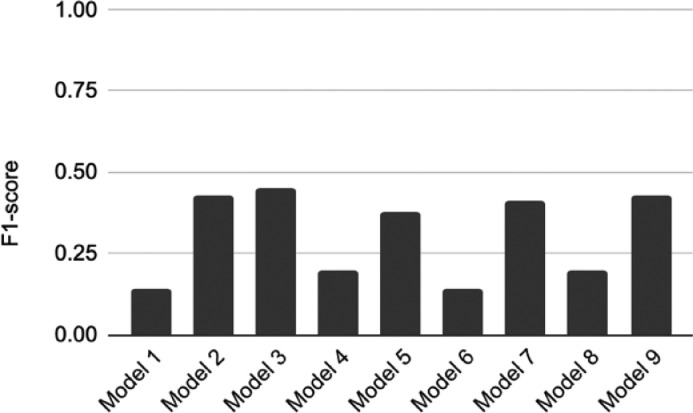
F1-score of all 9 Models.

The accuracy, AUROC, recall, precision, and F1 score were used in this present work to evaluate the prediction performance. Accuracy is the number of correct predictions over all data points. Precision is the positive predictive value, while recall is also known as sensitivity. High precision means the ability to return all the relevant cases, and a high recall means the ability to identify only the relevant data points The authors utilized F1-score as the main performance metric due to the unbalanced nature of the problem (there are more negative blood cultures than positive blood cultures). F1-score combines the recall and precision of a classifier into a single metric. F1-score will be low if either precision or recall is low. None of the models achieved an F1 score greater than 0.5 (Fig. 1).

## Results

Between 2018 and 2021, 2641 patients were admitted to the neonatal intensive care unit. The authors identified a total of 1181 blood cultures with paired Complete Blood Count and C-reactive protein collected at the same time. Fourteen samples were excluded due to missing data. Univariate analyses for blood culture positivity are presented in Table 1. Patients with positive blood cultures had lower hemoglobin, lymphocytes, and platelet levels, and higher neutrophils left shift and CRP levels (Table 1).

**Table 1:** Comparison between CBC and CRP values and blood culture positivity in neonates, univariate analysis.

Parameter	Negative blood culture (n = 1006)	Positive blood culture (n = 175)	p-value
Hemoglobin (g/dL), median (IQR)	13 (10.9–15.6)	11.6 (10–13.1)	**<0.001**^a^
Hematocrit (%), median (IQR)	37.6 (31.8–45.2)	33.3 (28.3–37)	**<0.001**^a^
MCV (fL), median (IQR)	100.4 (91.8–109.4)	94 (86.2–103.1)	**<0.001**^a^
MCH (pg) median (IQR)	34.8 (31.5–37.3)	32.5 (30.4–34.9)	**<0.001**^a^
MCHC (g/dL), median (IQR)	34.2 (33.2–35.6)	34.7 (33.6–36.3)	**0.005**^a^
Leukocytes, absolute count, median (IQR)	11070 (7490–16130)	12650 (7830–18610)	0.059^a^
Neutrophils (%), median (IQR)	53 (39.3–65)	54 (40–72)	0.070^a^
Neutrophils, absolute count, median (IQR)	5704 (3005–9450)	6663 (3276–11255)	**0.044**^a^
Left shift (%), median (IQR)	1 (0–4)	3 (0–9)	**<0.001**^a^
Left shift, absolute count, median (IQR)	69.3 (0–543)	350.4 (0–1234)	**<0.001**^a^
NLR, median (IQR)	1.58 (0.87–2.76)	1.93 (0.97–3.75)	**0.009**^a^
MLR, median (IQR)	0.28 (0.17–0.5)	0.38 (0.2–0.68)	**<0.001**^a^
PLR, median (IQR)	0.06 (0.04–0.09)	0.04 (0.02–0.08)	**<0.001**^a^
DNI, median (IQR)	0.02 (0–0.08)	0.06 (0–0.15)	**<0.001**^a^
Eosinophils (%), median (IQR)	2 (0.7–4)	2 (0.1–4.7)	0.941^a^
Eosinophils, absolute count, median (IQR)	185.2 (49.2–448.8)	180.3 (17.8–547)	0.963^a^
Basophils (%), median (IQR)	0.2 (0–0.8)	0 (0–0.9)	0.092^a^
Basophils, absolute count, median (IQR)	20.6 (0–73.2)	0 (0–63.75)	**0.041**^a^
Lymphocytes (%), median (IQR)	33 (22.9–45)	30 (18–41.3)	**<0.001**^a^
Lymphocytes, absolute count, median (IQR)	3448 (2219–4969)	3036 (2047–4415)	**0.031**^a^
Monocytes (%), median (IQR)	9.3 (6–13)	10 (6–16.8)	0.320^a^
Monocytes, absolute count, median (IQR)	1022 (573–1710)	1127 (582–1959)	0.079^a^
CRP (mg/L), median (IQR)	2.9 (0.7–10.9)	16.3 (2.9–46.2)	**<0.001**^a^
Platelet (K/mm^3^), median (IQR)	204 (127–291)	121 (69–234)	**<0.001**^a^
Lymphocyte to CRP ratio, median (IQR)	1238 (277–4462)	178 (60.5–1486)	**<0.001**^a^

a Mann-Whitney test. MCV, Mean Corpuscular Volume; MCH, Mean Corpuscular Hemoglobin, MCHC, Mean Corpuscular Hemoglobin Concentration; NLR, Neutrophil/Lymphocyte Ratio; MLR, Monocyte/Lymphocyte Ratio; PLR, Platelet/Lymphocyte Ratio; DNI, Delta Neutrophil Index; CRP, C-Reactive Protein.

In Model 1, the Random Forest Classifier achieved the highest accuracy (0.858) with an AUC of 0.767 in the training phase. After hyperparameter tuning, the model achieved an accuracy of 0.864, AUC of 0.765, Recall of 0.08, Precision of 1.00, and F1-score of 0.142 (Table 2). In Model 2 (Supplementary Table 1), the Extra Trees Classifier achieved the highest accuracy (0.856) with an AUC of 0.728 in the training phase. After hyperparameter tuning, the model achieved an accuracy of 0.774, AUC of 0.760, Recall of 0.596, Precision of 0.344, and F1-score of 0.436. In Model 3, the Extra Trees Classifier achieved the highest accuracy (0.859) with an AUC of 0.737 in the training phase. After hyperparameter tuning, the predictions made by the model in predicting bacteremia achieved an accuracy of 0.791, AUC of 0.775, Recall of 0.59, Precision of 0.36, and F1-score of 0.455 (Supplementary Table 2). In Model 4, the Linear Discriminant Analysis achieved the highest accuracy (0.856) with an AUC of 0.733. After hyperparameter tuning, the predictions made by the model in predicting bacteremia achieved an accuracy of 0.845, AUC of 0.733, Recall of 0.129, Precision of 0.628, and F1-score of 0.205 (Supplementary Table 3). In Model 5, the Extra Tree Classifier achieved the highest accuracy (0.863) with an AUC of 0.747. After hyperparameter tuning, the predictions made by the model in predicting bacteremia achieved an accuracy of 0.732, AUC of 0.748, Recall of 0.57, Precision of 0.29, and F1-score of 0.38 (Supplementary Table 4).

**Table 2:** Model 1 including all 25 parameters.

Model	Accuracy	AUROC	Recall	Precision	F1-score
Random Forest Classifier	0.858	0.767	0.128	0.57	0.20
Extra Trees Classifier	0.856	0.733	0.097	0.66	0.16
Logistic Regression	0.852	0.716	0.121	0.47	0.17
Ridge Classifier	0.851	0.000	0.081	0.47	0.13
Linear Discriminant Analysis	0.851	0.718	0.154	0.55	0.22
Light Gradient Boosting Machine	0.851	0.743	0.178	0.47	0.25
Gradient Boosting Classifier	0.849	0.746	0.227	0.51	0.30
Extreme Gradient Boosting	0.845	0.709	0.146	0.48	0.21
K Neighbors Classifier	0.839	0.630	0.064	0.30	0.10
Ada Boost Classifier	0.835	0.702	0.228	0.41	0.28
Decision Tree Classifier	0.797	0.586	0.285	0.35	0.30
Naïve Bayes	0.793	0.706	0.381	0.34	0.35
SVM – Linear Kernel	0.783	0.000	0.234	0.24	0.22
Quadratic Discriminant Analysis	0.686	0.612	0.462	0.25	0.32

SVM, Support Vector Machine; AUROC, Area Under the Receiver Operating Characteristic.

The authors identified 1911 blood cultures with paired CBC collected at the same time. Univariate analyses for blood culture positivity are presented in Table 3.

**Table 3:** Comparison between CBC parameters and blood culture positivity in neonates, univariate analysis.

Parameter	Negative blood culture (n = 1585)	Positive blood culture (n = 326)	p-value
Hemoglobin (g/dL), median (IQR)	12.8 (10.7–15.4)	11.3 (9.8–12.9)	**<0.001**^a^
Hematocrit (%), median (IQR)	37.3 (31.6–44.9)	32.7 (28.5–37)	**<0.001**^a^
MCV (fL), median (IQR)	100 (90.4–109.4)	92.7 (85.9–102.6)	**<0.001**^a^
MCH (pg), median (IQR)	34.5 (30.7–37.3)	32.1 (29.8–34.9)	**<0.001**^a^
MCHC (g/dL), median (IQR)	34.2 (33–35.4)	34.4 (33.3–35.9)	**0.007**^a^
Leukocytes, absolute count, median (IQR)	11220 (7440–16030)	13175 (8030–18880)	**<0.001**^a^
Neutrophils (%), median (IQR)	52 (38.4–64)	55 (40.4–72)	**<0.001**^a^
Neutrophils, absolute count, median (IQR)	5519 (2956–9104)	7125 (3314–11890)	**<0.001**^a^
Left shift (%), median (IQR)	1 (0–4)	3 (0–8)	**<0.001**^a^
Left shift, absolute count, median (IQR)	91.1 (0–545.1)	342 (0–1206)	**<0.001**^a^
NLR, median (IQR)	1.5 (0.84–2.67)	1.95 (1–4.05)	**<0.001**^a^
MLR, median (IQR)	0.27 (0.16–0.48)	0.375 (0.18–0.64)	**<0.001**^a^
PLR, median (IQR)	0.06 (0.04–0.09)	0.04 (0.02–0.07)	**<0.001**^a^
DNI, median (IQR)	0.02 (0–0.09)	0.05 (0–0.13)	**<0.001**^a^
Eosinophils (%), median (IQR)	2 (0.8–4.4)	2 (0.4–4.1)	0.315^a^
Eosinophils, absolute count, median (IQR)	194.6 (52.4–515.1)	193 (30–583)	0.665^a^
Basophils (%), median (IQR)	0.2 (0–0.8)	0 (0–0.7)	**0.003**^a^
Basophils, absolute count, median (IQR)	9.96 (0–73.1)	0 (0–59.1)	**0.002**^a^
Lymphocytes (%), median (IQR)	34.2 (23.2–46)	28.7 (17–41)	**<0.001**^a^
Lymphocytes, absolute count, median (IQR)	3556 (2322–5117)	3141 (2047–4704)	**0.005**^a^
Monocytes (%), median (IQR)	9 (6–13)	9 (5–15.3)	0.810^a^
Monocytes, absolute count, median (IQR)	985 (557–1661)	121 (50–226)	0.010^a^
Platelet (K/mm^3^), median (IQR)	203 (127–292)	121 (50–226)	**<0.001**^a^

a Mann-Whitney test. MCV, Mean Corpuscular Volume; MCH, Mean Corpuscular Hemoglobin; MCHC, Mean Corpuscular Hemoglobin Concentration; NLR, Neutrophil/Lymphocyte Ratio; MLR, Monocyte/Lymphocyte Ratio; PLR, Platelet/Lymphocyte Ratio; DNI, Delta Neutrophil Index.

In Model 6, the Gradient Boosting Classifier achieved the highest accuracy (0.848) with an AUC of 0.737. After hyperparameter tuning, the predictions made by the model in predicting bacteremia achieved an accuracy of 0.808, AUC of 0.727, Recall of 0.08, Precision of 0.56, and F1-score of 0.14 (Supplementary Table 5). In Model 7, the Extra Trees Classifier achieved the highest accuracy (0.844) with an AUC of 0.726. After hyperparameter tuning, the predictions made by the model in predicting bacteremia achieved an accuracy of 0.688, AUC of 0.706, Recall of 0.56, Precision of 0.32, and F1-score of 0.41 (Supplementary Table 6). In Model 8, the Linear Discriminant Analysis achieved the highest accuracy (0.848) with an AUC of 0.742. After hyperparameter tuning, the predictions made by the model in predicting bacteremia achieved an accuracy of 0.803, AUC of 0.695, Recall of 0.12, Precision of 0.48, and F1-score of 0.19 (Supplementary Table 7). In Model 9, the Extra Trees Classifier achieved the highest accuracy (0.849) with an AUC of 0.744. After hyperparameter tuning, the predictions made by the model in predicting bacteremia achieved an accuracy of 0.716, AUC of 0.714, Recall of 0.56, Precision of 0.35, and F1-score of0.43 (Supplementary Table 8).

## Discussion

The present study shows that ML models based on CBC and CRP cannot be used to predict neonatal bacteremia in routine clinical practice in the neonatal intensive care unit. Although the models had a reasonable accuracy (0.688–0.864) and AUROC (0.695–0.765), the prediction of bacteremia is an unbalanced situation, where there are significantly more negative blood cultures than positive ones. Recall, precision, and F1-score are superior metrics to assess ML classification performance in unbalanced problems. All of the models showed poor recall, precision, and F1-score Table 4.

**Table 4:** Summary of metrics for the 9 ML models.

	Best Algorithm	Accuracy	AUROC	Recall	Precision	F1
**Model 1**	Random Forest Classifier	0.864	0.765	0.08	1.00	0.14
**Model 2**	Extra Trees Classifier	0.774	0.760	0.59	0.34	0.43
**Model 3**	Extra Trees Classifier	0.791	0.775	0.59	0.36	0.45
**Model 4**	Linear Discriminant Analysis	0.845	0.743	0.13	0.41	0.20
**Model 5**	Extra Trees Classifier	0.732	0.748	0.57	0.29	0.38
**Model 6**	Gradient Boosting Classifier	0.808	0.727	0.08	0.56	0.14
**Model 7**	Extra Trees Classifier	0.688	0.706	0.56	0.32	0.41
**Model 8**	Linear Discriminant Analysis	0.803	0.695	0.12	0.48	0.19
**Model 9**	Extra Trees Classifier	0.716	0.714	0.56	0.35	0.43

Bacteremia is a potentially fatal condition that requires early diagnosis and prompt treatment. Laboratory biomarkers have been widely examined to detect early bacteremia due to challenges in evaluating blood culture, which are the gold standard and the fact that signs and symptoms of neonatal sepsis might be subtle and challenging to interpret. Unfortunately, there are still no diagnostic biomarkers that can determine whether or not to withhold antibiotics.

Two major objectives of clinical research are inference and prediction. To understand or test a hypothesis, inference is crucial. Statistics uses a sample to draw inferences about the population. Without knowing the underlying mechanism, prediction aims to foresee outcomes. Generalizable predictive patterns are discovered using machine learning. Both inference and prediction are significant in clinical research. The authors are interested in both the whys of biological processes and their future developments.^11^ Due to growing computer power and the massive healthcare data generation, machine learning is now increasingly frequently employed to predict outcomes in medicine.^12^

The authors tested several feature selection methods, machine learning algorithms, and hyperparameter tweaking techniques, without being able to build a high-performing ML model. It is likely that the selected variables (CBC and CRP values) are insufficient to predict positive blood culture in neonates. Even though artificial intelligence and machine learning are revolutionizing healthcare, if the correct variables are not incorporated into ML models predictions will be poor. Thus, it is not that biomarkers are not useful to predict bacteremia; rather, CBC and CRP are the incorrect biomarkers for this purpose. As a result, the authors recommend that novel biomarkers be investigated in machine learning models instead of using CBC and CRP to predict a positive blood culture in newborns. In a study by Boerman et al., the authors analyzed machine learning to predict blood culture outcomes^13^ in the emergency department. The authors found a similar AUROC to the present study (0.77‒0.78) even using demographic data as parameters. However, the F1-score was very low (0.14–0.17).

It is important to note the limitations of the study. First, although it is a frequent contaminant, Coagulase-negative Staphylococcus can be a pathogen in newborns. The authors included all blood cultures positive for Coagulase-negative Staphylococci because it can be challenging to distinguish between contamination and true infection in neonates. Second, the decision to take blood cultures was dependent on the attending physician's clinical assessment, so patient heterogeneity may be taken into account. Those variables can be controlled in a prospective study. Third, the scope of this investigation was restricted to looking only into bacterial bloodstream infections. It is important to note that the authors did not include demographic and clinical data as parameters in the prediction models.

## Conclusion

In conclusion, this study has demonstrated that it is not possible to predict bacteremia in neonates using ML models based on CBC and CRP. Other biomarkers should be evaluated in machine-learning models to predict bloodstream infections in neonates.

## Authors’ contributions

Matsushita FY contributions: Conceptualization, Data curation, Formal analysis, Investigation, Methodology, Software, Validation, Writing - original-draft. Krebs VLJ and Carvalho WB attributions: Conceptualization, Project administration, Supervision, Validation, Writing - review & editing.

## Conflicts of interest

The authors declare no conflicts of interest.

## Appendix Supplementary materials

Image, application 1
