# Supplementary material for: Complete blood count and C-reactive protein to predict positive blood culture among neonates using machine learning algorithms
Source: Clinics (Sao Paulo). 2022 Dec 8;78:100148. doi: 10.1016/j.clinsp.2022.100148 (PMC9763374; doi:10.1016/j.clinsp.2022.100148)
Supplement: Supplementary file 1 [file mmc1.docx]

**CLINICS-D-22-00407 – Supplementary Material**

**Supplementary Table 1**. Model 2 using only variables that were statistically significant for positive blood culture in univariate analysis.

| **Model** | **Accuracy** | **AUROC** | **Recall** | **Precision** | **F1-score** |
| --- | --- | --- | --- | --- | --- |
| Extra Trees Classifier | 0.8560 | 0.7280 | 0.1218 | 0.5200 | 0.1956 |
| Random Forest Classifier | 0.8548 | 0.7437 | 0.1622 | 0.5500 | 0.2448 |
| Ridge Classifier | 0.8500 | 0.0000 | 0.0744 | 0.5250 | 0.1229 |
| Gradient Boosting Classifier | 0.8499 | 0.7416 | 0.2109 | 0.4886 | 0.2877 |
| Logistic Regression | 0.8475 | 0.7095 | 0.0897 | 0.5076 | 0.1423 |
| Light Gradient Boosting Machine | 0.8475 | 0.7446 | 0.1788 | 0.5467 | 0.2596 |
| Linear Discriminant Analysis | 0.8464 | 0.7154 | 0.1391 | 0.5875 | 0.2145 |
| K Neighbors Classifier | 0.8426 | 0.6628 | 0.1295 | 0.4319 | 0.1945 |
| Extreme Gradient Boosting | 0.8402 | 0.7148 | 0.1705 | 0.4170 | 0.2360 |
| Ada Boost Classifier | 0.8391 | 0.7197 | 0.2365 | 0.3978 | 0.2934 |
| Naïve Bayes | 0.8015 | 0.7091 | 0.3321 | 0.3549 | 0.3301 |
| Quadratic Discriminant Analysis | 0.7857 | 0.6553 | 0.3571 | 0.3999 | 0.3488 |
| Decision Tree Classifier | 0.7844 | 0.5717 | 0.2686 | 0.2555 | 0.2568 |
| SVM – Linear Kernel | 0.7831 | 0.0000 | 0.2353 | 0.3238 | 0.2345 |

SVM, Support Vector Machine; AUROC, Area Under the Receiver Operating Characteristic.

**Supplementary Table 2** Model 3 using Boruta SHAP as feature selection algorithm.

| **Model** | **Accuracy** | **AUROC** | **Recall** | **Precision** | **F1-score** |
| --- | --- | --- | --- | --- | --- |
| Extra Trees Classifier | 0.8596 | 0.7374 | 0.1218 | 0.6333 | 0.1985 |
| Logistic Regression | 0.8548 | 0.7361 | 0.1135 | 0.5817 | 0.1811 |
| Ridge Classifier | 0.8536 | 0.0000 | 0.0814 | 0.4750 | 0.1311 |
| Linear Discriminant Analysis | 0.8524 | 0.7433 | 0.1468 | 0.7125 | 0.2301 |
| Random Forest Classifier | 0.8499 | 0.7414 | 0.1545 | 0.5333 | 0.2288 |
| K Neighbors Classifier | 0.8451 | 0.6625 | 0.1071 | 0.4100 | 0.1618 |
| Gradient Boosting Classifier | 0.8451 | 0.7188 | 0.1795 | 0.4508 | 0.2548 |
| Light Gradient Boosting Machine | 0.8439 | 0.7418 | 0.1865 | 0.4081 | 0.2495 |
| Ada Boost Classifier | 0.8415 | 0.7191 | 0.2692 | 0.4426 | 0.3295 |
| Extreme Gradient Boosting | 0.8342 | 0.7306 | 0.1628 | 0.3989 | 0.2232 |
| Quadratic Discriminant Analysis | 0.8232 | 0.6955 | 0.3013 | 0.4202 | 0.3379 |
| Naive Bayes | 0.8196 | 0.7080 | 0.3167 | 0.4073 | 0.3447 |
| SVM – Linear Kernel | 0.8099 | 0.0000 | 0.2801 | 0.3478 | 0.2918 |
| Decision Tree Classifier | 0.7834 | 0.5796 | 0.2885 | 0.2827 | 0.2734 |

SVM, Support Vector Machine; AUROC, Area Under the Receiver Operating Characteristic.

**Supplementary Table 3** Model 4 using features according to expert’s opinion.

| **Model** | **Accuracy** | **AUROC** | **Recall** | **Precision** | **F1-score** |
| --- | --- | --- | --- | --- | --- |
| Linear Discriminant Analysis | 0.8560 | 0.7331 | 0.1295 | 0.6283 | 0.2055 |
| Logistic Regression | 0.8536 | 0.7327 | 0.0897 | 0.5833 | 0.1472 |
| Ridge Classifier | 0.8512 | 0.0000 | 0.0744 | 0.4750 | 0.1218 |
| K Neighbors Classifier | 0.8511 | 0.6681 | 0.1301 | 0.5583 | 0.2035 |
| Quadratic Discriminant Analysis | 0.8488 | 0.7206 | 0.1795 | 0.5568 | 0.2592 |
| Random Forest Classifier | 0.8487 | 0.7184 | 0.2045 | 0.4621 | 0.2799 |
| Ada Boost Classifier | 0.8427 | 0.6831 | 0.1718 | 0.4350 | 0.2454 |
| SVM – Linear Kernel | 0.8414 | 0.0000 | 0.1160 | 0.3729 | 0.1613 |
| Extra Trees Classifier | 0.8378 | 0.7051 | 0.1551 | 0.3915 | 0.2175 |
| Gradient Boosting Classifier | 0.8366 | 0.7099 | 0.1474 | 0.3831 | 0.2096 |
| Naïve Bayes | 0.8342 | 0.7314 | 0.2186 | 0.4449 | 0.2837 |
| Extreme Gradient Boosting | 0.8232 | 0.6640 | 0.2186 | 0.3644 | 0.2694 |
| Light Gradient Boosting Machine | 0.8221 | 0.6849 | 0.1865 | 0.3376 | 0.2361 |
| Decision Tree Classifier | 0.7844 | 0.5855 | 0.3019 | 0.2844 | 0.2895 |

SVM, Support Vector Machine; AUROC, Area Under the Receiver Operating Characteristic.

**Supplementary Table 4** Model 5 using PyCaret’s feature_selection.

| **Model** | **Accuracy** | **AUROC** | **Recall** | **Precision** | **F1** |
| --- | --- | --- | --- | --- | --- |
| Extra Trees Classifier | 0.8632 | 0.7475 | 0.1212 | 0.6250 | 0.1990 |
| Logistic Regression | 0.8524 | 0.7224 | 0.1212 | 0.4562 | 0.1775 |
| Random Forest Classifier | 0.8523 | 0.7569 | 0.1301 | 0.5533 | 0.2035 |
| Gradient Boosting Classifier | 0.8511 | 0.7457 | 0.1949 | 0.5239 | 0.2804 |
| Ridge Classifier | 0.8500 | 0.0000 | 0.0814 | 0.4650 | 0.1296 |
| Linear Discriminant Analysis | 0.8475 | 0.7293 | 0.1385 | 0.5107 | 0.2023 |
| K Neighbors Classifier | 0.8451 | 0.6250 | 0.1045 | 0.4686 | 0.1645 |
| Ada Boost Classifier | 0.8451 | 0.7053 | 0.2769 | 0.4784 | 0.3486 |
| Light Gradient Boosting Machine | 0.8451 | 0.7566 | 0.1551 | 0.4250 | 0.2229 |
| Extreme Gradient Boosting | 0.8402 | 0.7325 | 0.1705 | 0.4626 | 0.2386 |
| SVM – Linear Kernel | 0.8076 | 0.0000 | 0.2013 | 0.2817 | 0.2196 |
| Naïve Bayes | 0.8014 | 0.7135 | 0.3801 | 0.3670 | 0.3666 |
| Decision Tree Classifier | 0.7820 | 0.5709 | 0.2699 | 0.2805 | 0.2643 |
| Quadratic Discriminant Analysis | 0.7541 | 0.6155 | 0.3974 | 0.3039 | 0.3342 |

SVM, Support Vector Machine; AUROC, Area Under the Receiver Operating Characteristic.

**Supplementary Table 5** Model 6 using all parameters without C-reactive protein.

| **Model** | **Accuracy** | **AUROC** | **Recall** | **Precision** | **F1-score** |
| --- | --- | --- | --- | --- | --- |
| Gradient Boosting Classifier | 0.8489 | 0.7378 | 0.2058 | 0.5932 | 0.3015 |
| Extra Trees Classifier | 0.8459 | 0.7430 | 0.0703 | 0.6717 | 0.1233 |
| Random Forest Classifier | 0.8452 | 0.7439 | 0.1591 | 0.5424 | 0.2420 |
| Linear Discriminant Analysis | 0.8422 | 0.7491 | 0.1452 | 0.5400 | 0.2236 |
| Logistic Regression | 0.8414 | 0.7471 | 0.1312 | 0.5167 | 0.2052 |
| Ridge Classifier | 0.8414 | 0.0000 | 0.0470 | 0.5000 | 0.0849 |
| Light Gradient Boosting Machine | 0.8400 | 0.7607 | 0.2149 | 0.5003 | 0.2972 |
| Extreme Gradient Boosting | 0.8325 | 0.7536 | 0.2152 | 0.4733 | 0.2879 |
| Ada Boost Classifier | 0.8303 | 0.7101 | 0.2056 | 0.4533 | 0.2768 |
| K Neighbors Classifier | 0.8220 | 0.6427 | 0.1452 | 0.3715 | 0.2059 |
| SVM – Linear Kernel | 0.8078 | 0.0000 | 0.3058 | 0.3528 | 0.3119 |
| Naïve Bayes | 0.8033 | 0.7292 | 0.2524 | 0.3562 | 0.2912 |
| Quadratic Discriminant Analysis | 0.8018 | 0.6959 | 0.2437 | 0.3251 | 0.2726 |
| Decision Tree Classifier | 0.7487 | 0.5668 | 0.2994 | 0.2550 | 0.2749 |

SVM, Support Vector Machine; AUROC, Area Under the Receiver Operating Characteristic.

**Supplementary Table 6** Model 7 using only variables that were statistically significant for positive blood culture in univariate analysis (excluding C-reactive protein).

| **Model** | **Accuracy** | **AUROC** | **Recall** | **Precision** | **F1-score** |
| --- | --- | --- | --- | --- | --- |
| Extra Trees Classifier | 0.8444 | 0.7261 | 0.0939 | 0.5488 | 0.1568 |
| Gradient Boosting Classifier | 0.8437 | 0.7375 | 0.1727 | 0.5269 | 0.2533 |
| Random Forest Classifier | 0.8429 | 0.7313 | 0.1548 | 0.5650 | 0.2374 |
| Ridge Classifier | 0.8392 | 0.0000 | 0.0374 | 0.5000 | 0.0693 |
| Logistic Regression | 0.8384 | 0.7416 | 0.0983 | 0.4600 | 0.1565 |
| Linear Discriminant Analysis | 0.8362 | 0.7450 | 0.1074 | 0.4400 | 0.1675 |
| Light Gradient Boosting Machine | 0.8362 | 0.7269 | 0.1866 | 0.4531 | 0.2558 |
| Extreme Gradient Boosting | 0.8295 | 0.7202 | 0.1963 | 0.4177 | 0.2631 |
| Ada Boost Classifier | 0.8213 | 0.6903 | 0.1874 | 0.3823 | 0.2472 |
| K Neighbors Classifier | 0.8190 | 0.6293 | 0.1411 | 0.2936 | 0.1896 |
| Naïve Bayes | 0.8070 | 0.7330 | 0.2290 | 0.3536 | 0.2736 |
| Quadratic Discriminant Analysis | 0.8063 | 0.7062 | 0.1641 | 0.2914 | 0.2080 |
| SVM – Linear Kernel | 0.7898 | 0.0000 | 0.2396 | 0.3016 | 0.2530 |
| Decision Tree Classifier | 0.7539 | 0.5703 | 0.3000 | 0.2662 | 0.2797 |

SVM, Support Vector Machine; AUROC, Area Under the Receiver Operating Characteristic.

**Supplementary Table 7** Model 8 using Boruta SHAP as feature selection algorithm (excluding C-reactive protein).

| **Model** | **Accuracy** | **AUROC** | **Recall** | **Precision** | **F1-score** |
| --- | --- | --- | --- | --- | --- |
| Linear Discriminant Analysis | 0.8489 | 0.7424 | 0.1310 | 0.7103 | 0.2117 |
| Logistic Regression | 0.8482 | 0.7395 | 0.1216 | 0.7025 | 0.2001 |
| Extra Trees Classifier | 0.8467 | 0.7479 | 0.1353 | 0.5455 | 0.2131 |
| Ridge Classifier | 0.8414 | 0.0000 | 0.0279 | 0.4000 | 0.0518 |
| Gradient Boosting Classifier | 0.8399 | 0.7229 | 0.1723 | 0.4940 | 0.2480 |
| Light Gradient Boosting Machine | 0.8399 | 0.7332 | 0.2139 | 0.4910 | 0.2941 |
| Random Forest Classifier | 0.8392 | 0.7400 | 0.1729 | 0.4962 | 0.2531 |
| Quadratic Discriminant Analysis | 0.8355 | 0.7400 | 0.1591 | 0.4728 | 0.2307 |
| K Neighbors Classifier | 0.8340 | 0.6573 | 0.1968 | 0.4559 | 0.2718 |
| Ada Boost Classifier | 0.8340 | 0.7011 | 0.2201 | 0.4783 | 0.2964 |
| Naïve Bayes | 0.8325 | 0.7394 | 0.1920 | 0.4843 | 0.2606 |
| Extreme Gradient Boosting | 0.8242 | 0.7253 | 0.1814 | 0.4087 | 0.2467 |
| SVM – Linear Kernel | 0.8086 | 0.0000 | 0.2043 | 0.2700 | 0.2058 |
| Decision Tree Classifier | 0.7682 | 0.5800 | 0.3035 | 0.2887 | 0.2914 |

SVM, Support Vector Machine; AUROC, Area Under the Receiver Operating Characteristic.

**Supplementary Table 8** Model 9 using PyCaret’s feature_selection (excluding C-reactive protein).

| **Model** | **Accuracy** | **AUROC** | **Recall** | **Precision** | **F1-score** |
| --- | --- | --- | --- | --- | --- |
| Extra Trees Classifier | 0.8497 | 0.7444 | 0.1173 | 0.6364 | 0.1917 |
| Random Forest Classifier | 0.8467 | 0.7551 | 0.1494 | 0.6122 | 0.2295 |
| Gradient Boosting Classifier | 0.8452 | 0.7410 | 0.1959 | 0.5536 | 0.2865 |
| Light Gradient Boosting Machine | 0.8445 | 0.7532 | 0.2242 | 0.5286 | 0.3093 |
| Logistic Regression | 0.8429 | 0.7469 | 0.1266 | 0.5357 | 0.2007 |
| Ridge Classifeir | 0.8429 | 0.0000 | 0.0470 | 0.5667 | 0.0859 |
| Linear Discriminant Analysis | 0.8422 | 0.7502 | 0.1452 | 0.5332 | 0.2227 |
| Extreme Gradient Boosting | 0.8370 | 0.7427 | 0.2290 | 0.5243 | 0.3102 |
| Ada Boost Classifier | 0.8235 | 0.7080 | 0.1913 | 0.3947 | 0.2518 |
| K Neighbors Classifier | 0.8227 | 0.6528 | 0.1225 | 0.3335 | 0.1726 |
| Quadratic Discriminant Analysis | 0.8115 | 0.7254 | 0.1597 | 0.3142 | 0.2106 |
| Naïve Bayes | 0.8078 | 0.7254 | 0.2385 | 0.3755 | 0.2869 |
| SVM – Linear Kernel | 0.8040 | 0.0000 | 0.2563 | 0.3205 | 0.2766 |
| Decision Tree Classifier | 0.7480 | 0.5649 | 0.2955 | 0.2569 | 0.2730 |

SVM, Support Vector Machine; AUROC, Area Under the Receiver Operating Characteristic.
